# Peptides for diagnosis and treatment of ovarian cancer

**DOI:** 10.3389/fonc.2023.1135523

**Published:** 2023-05-05

**Authors:** Ling Guo, Jing Wang, Nana Li, Jialin Cui, Yajuan Su

**Affiliations:** Department of Clinical Laboratory, Harbin Medical University Cancer Hospital, Harbin, China

**Keywords:** peptides, ovarian cancer, diagnosis, targeted therapy, peptide vaccine

## Abstract

Ovarian cancer is the most deadly gynecologic malignancy, and its incidence is gradually increasing. Despite improvements after treatment, the results are unsatisfactory and survival rates are relatively low. Therefore, early diagnosis and effective treatment remain two major challenges. Peptides have received significant attention in the search for new diagnostic and therapeutic approaches. Radiolabeled peptides specifically bind to cancer cell surface receptors for diagnostic purposes, while differential peptides in bodily fluids can also be used as new diagnostic markers. In terms of treatment, peptides can exert cytotoxic effects directly or act as ligands for targeted drug delivery. Peptide-based vaccines are an effective approach for tumor immunotherapy and have achieved clinical benefit. In addition, several advantages of peptides, such as specific targeting, low immunogenicity, ease of synthesis and high biosafety, make peptides attractive alternative tools for the diagnosis and treatment of cancer, particularly ovarian cancer. In this review, we focus on the recent research progress regarding peptides in the diagnosis and treatment of ovarian cancer, and their potential applications in the clinical setting.

## 1 Introduction

Ovarian cancer (OC) is a common gynecologic malignancy with 314,000 new cases and 207,000 deaths in 2020 year (1). Due to the early stage with unique symptoms such as pelvic/abdominal pain, abdominal distension, and frequent urination/urgency, and the lack of apparent diagnostic indicators, more than 70% of patients had advanced disease at presentation (2). OC treatment regimens are generally platinum- and paclitaxel-based chemotherapy regimens after initial cytoreductive surgery (3). Despite improvement after treatment, most patients experience relapse within 3 years due to the development of chemoresistance, leading to a significant decrease in survival (4). In addition, due to the lack of targeting, chemotherapy drugs not only kill cancer cells but also damage normal cells, which has certain toxic side effects on patients. Therefore, the development of specific early diagnostic indicators and the improvement of anti-tumor drug targeting are two issues that need to be addressed urgently.

The development of targeted therapy, including targeted anti-angiogenesis therapy, immune checkpoint inhibitor therapy and Poly-ADP-ribose polymerase (PARP) inhibitors therapy, brings hope to many OC patients (5). In targeted therapy, the selection of appropriate ligands is a crucial step. In the past, antibody-drug conjugate (ADC) was an advanced targeted drug delivery strategy for OC therapy, which could selectively deliver cytotoxic drugs to tumor cells. However, its application *in vivo* was limited by poor delivery due to the high molecular weight of antibodies and systemic toxicity caused by non-specific uptake of the reticuloendothelial system (6). In recent years, peptides have attracted a lot of attention in tumor diagnosis and treatment. They are naturally occurring compounds, usually 2-50 amino acid residues (AA) long, responsible for many biological functions. As targeting ligands, peptides exhibit high affinity and specificity for the corresponding receptors. Compared with antibodies and proteins, polypeptides have the characteristics of small molecular weight, low immunogenicity, and easy chemical modification, which makes the polypeptide have stronger cell, tissue penetration and metabolic stability (7). Moreover, combinatorial library chemistry and phage display strategies provide source safeguards for the development of various peptides with targeting potential (8).

Currently, peptide-based research is attracting numerous researchers for development and application. In addition to its use as a targeting ligand for therapeutic purposes it is also (I) widely used in diagnostic imaging due to its specific targeting and rapid renal clearance (9); (II) differential peptides secreted in body fluids are used as diagnostic markers (10); (III) some peptides are related to cancer cell growth, migration, invasion and other activities, and these differentially expressed or peptides acting on therapeutic targets inhibit the occurrence and development of tumors (11); (IV) peptide vaccines (12). Based on these properties, peptides may be promising candidates for tumor diagnosis and therapy. Some excellent reviews have reported the value of peptides in cancer diagnosis and treatment (13, 14); Here, we discuss the role of peptides in the diagnosis and treatment of ovarian cancer.

## 2 Peptide diagnosis in ovarian cancer

Peptide biomarkers can be widely divided into laboratory diagnosis and imaging diagnosis in diagnostic methods. In terms of laboratory diagnosis, some endogenous peptides can reveal the different pathology and heterogeneity of diseases by reflecting the expression of disease proteins or changes in the corresponding enzyme activities, which have the potential to become promising diagnostic markers (15). In terms of imaging, peptide receptors overexpressed in ovarian cancer have been used as molecular targets to localize tumors, and radiolabeled peptides can accurately localize primary tumor lesions (16).

### 2.1 Peptide-based laboratory diagnosis

In clinical laboratory diagnosis, the research focus is still on biological specimens such as blood, urine, saliva, and cerebrospinal fluid, which can be obtained by non-invasive or minimally invasive techniques (17). In general, abnormal proteins in body fluids such as CA125 and HE4 have been the first choice for ovarian cancer examination. However, detection sensitivity and specificity continue to fall short of the desired aim, and there are certain limits in early diagnosis (18). The exploration of peptide markers has attracted much attention in recent years and has potential clinical application prospects. Peptide markers can be relatively resistant to protease degradation, and small peptides may survive even if the protein is degraded by proteases in the tumor extracellular space or circulation (19). Therefore, it may provide more information than the precursor protein (Protein containing the target polypeptide sequence). Moreover, advances in technologies such as peptidomics, peptide microarray, and reaction monitoring (SRM) analysis support the detection of more low-molecular-weight, tumor-specific peptides in body fluids. In addition to high specificity, SRM analysis provides sensitivity and linearity, providing an opportunity for accurate quantification of peptides (20, 21).

The research has shown that peptides with different clinical features can be identified, quantified and compared using detection techniques, and these low molecular weight peptides are more likely to be markers for diagnosis. A prospective study about urinary micropeptides in patients with ovarian cancer has shown that they presenced in about 62.5% of patients, and were related to the pathological stage. Under 100% specificity, the detection sensitivity of CA125 was 23.2%, while that of urinary micropeptides was 62.5% (22). At the peptidome level, more peptides could be observed in serum samples compared to plasma. However, serum shows many strong artificial peptide signals that hinder the sensitive detection of endogenous peptides. Therefore, most investigators have turned their goals to plasma samples (23, 24). Wang and colleagues (20) used SRM method to detect peptide biomarkers and found that 11 (64.7%) of 17 patients with early ovarian cancer had positive scores of small peptides from peptidyl prolyl cis-trans isomerase A(PPIA), which may be a useful diagnostic marker for ovarian cancer. Lu’s group (18) isolated three promising predictive small peptides from plasma samples of 140 patients with epithelial ovarian cancer (EOC) and 158 patients with benign ovarian tumors (BOT), and their combination with CA125 increased the specificity (0.944) and AUC (0.904) of CA125 while maintaining an acceptable sensitivity of 0.804.

### 2.2 Peptide-based imaging diagnosis

Traditional imaging techniques rely on non-specific imaging methods for disease examination, such as image contrast using anatomical, physiological, or metabolic abnormalities, and are often less efficient at targeting (25). In fact, molecular or biochemical alterations in disease often precede morphological and functional alterations and can more accurately characterize tumor properties or biological processes (26). Based on the observation that peptides can bind specifically to disease-associated receptors, coupling imaging molecules to peptides is a simple and accurate way to improve traditional imaging. In general, the size of the polypeptide can provide sufficient attachment sites for the chelating agent molecule, which is more suitable for linking with the chelating agent than the larger molecule, without losing the binding affinity with the receptor (27). The advantages of using radiopeptide imaging include the ease of synthesis and radiolabeling, as well as the ease of modification that enhances renal excretion and significantly improves kinetics (28). Furthermore, the small molecular weight of peptide allows for rapid blood clearance and renal excretion, which makes it among the key features of an ideal imaging probe. Many selected peptides or peptide analogues used to target OC-related molecules or receptors show high affinity and specificity for the receptors, such as RGD, NGR, etc (**Table 1**) (37, 38).

**Table 1:** Peptide ligands for imaging diagnostics.

Peptide sequence	Tagert	Probes	Tumor/Background	Application Phase	Ref.
RGD	αvβ3	^111^In-DOTA-E-[c(RGDfK)]_2_	96 (Tumor/Blood)	Experimental Phase	(29)
		^18^F-RGD_2_	6.04 ± 0.92	Experimental Phase	(30)
		^18^F-FPPRGD_2_	3.7 ± 1.3	Clinical Phase	(31)
NGR	CD13	Cy5.5-NGR-Fe_3_O_4_-NPs	–	Experimental Phase	(32)
		68Ga-DOTA-c(NGR)_2_	10.30 ± 0.26	Experimental Phase	(33)
GE137	c-Met	Cy5- GE137	–	Experimental Phase	(34)
GnRH	GnRHR	GnRH-ICG	7.41 ± 2.82 (2H)	Experimental Phase	(35)
FSHβ	FSHR	DCNPs-Cy7-FSHβ	12.5(20H)	Experimental Phase	(36)

“-”: Not mentioned in the text.

The RGD (Arg-Gly-Asp) peptide recognizes the highly expressed α_v_β_3_ integrin in ovarian cancer cells and can specifically bind α_v_β_3_ with high affinity after radiolabeling. Imaging probes based on RGD peptides show good imaging effects on ovarian cancer and are one of the most promising diagnostic probes (39). Janssen and colleagues (29) synthesized radiolabeled dimeric RGD peptide E- [c (RGDfK)]_2_, which showed specific targeting, good tumor uptake, and rapid renal excretion in the OVCAR-3 xenograft model. Yang’s group (30)found that ^18^F-RGD2 had a higher tumor/background ratio than ^18^F-FDG in SKOV-3 xenograft mice (6.04 ± 0.92 vs. 3.34 ± 0.79), and in addition, the anti-angiogenic effect was sensitively and reliably detected at low doses of paclitaxel. At present, imaging probes based on RGD peptides have entered preclinical or clinical trials. In a clinical study of ovarian and breast cancer, the PET/CT imaging analysis found that RGD-based imaging probes (^18^F-FPPRGD_2_) can provide independent information with ^18^F-FDG, although the foci uptake and tumor/background ratio of ^18^F-FPPRGD_2_ were lower than ^18^F-FDG, but 1 patient increased uptake after 1 week of anti-angiogenesis therapy. This is a potential benefit of RGD peptide-based PET tracers with the potential to predict anti-angiogenesis therapy early (31).

Aminopeptidase N (APN/CD13), a membrane protein and marker of tumor angiogenesis, is upregulated in various solid tumors, including melanoma, prostate, ovarian, lung, and breast cancers (40). NGR (Asn-Gly-Arg) peptide specifically recognizes aminopeptidase N on the surface of tumor cells. Radiolabeled NGR has been reported to have high uptake in ovarian tumors and is excreted through the kidneys (41). Meng and colleagues (32)prepared Cy5.5-labeled, NGR-conjugated iron oxide nanoparticles (Cy5.5-NGR-Fe_3_O_4_ NPs) as imaging nanoprobe. MR and NIRF dual-modality imaging showed that NGR targeting effectively improved tumor uptake and enhanced imaging contrast, in contrast, the control group image did not change significantly. In another work, NGR peptide-based imaging probes exhibited selective targeting and quick blood clearance of ES2 tumors, with high contrast tumor visualization occurring at 1 h and a maximal tumor/background ratio of 10.30 ± 0.26 (33).

In addition to radiolabeling, optical imaging of fluorescently labeled peptides may be advantageous because it may improve the detection of metastatic tumor deposits while reducing the risk of population radioactivity exposure (42, 43). Liu and colleagues (34) report the biological characterization of fluorescently labeled GE137 (c-Met targeting peptide) in ovarian cancer cells. The peptide demonstrated selective tumor uptake and a consistently high signal-to-noise ratio, allowing real-time observation of subcutaneous submillimeter SKOV3 tumor deposition in mice. Besides, some fluorescent probes can be used to detect ovarian cancer metastasis. In ovarian cancer mice, Liu’s group found that GnRHa-ICG was specifically localized in peritoneal tumors with high tumor-to-muscle and tumor-to-gut ratios and stable fluorescent signals compared to ICG injection alone (35). Intraoperative specific fluorescence imaging has been reported to improve tumor stage and size and improve prognosis. Wang’s team (36) designed *in vivo* assembly of NIR-II emitting down conversion nanoparticles (DCNPs) modified with DNA and FSHβ peptides. Imaging showed that the tumor background ratio remained at 12.5 for 20-28 hours after injection, the images could clearly distinguished the tumor margin, and HE staining demonstrated that even macroscopically invisible ultra-small tumors ≤ 1 mm could be detected and completely resected.

## 3 Peptide therapy in ovarian cancer

Peptide-based therapy can be divided into three main types, peptide ligand targeted therapy, anticancer peptide therapy, and peptide vaccines. Peptides are employed as ligands for targeted treatment, recognizing tumor markers and the ability to effectively deliver therapeutic drugs to tumor sites to enhance efficacy and minimize side effects (44). Anticancer peptides are proliferation inhibitors that exhibit antitumor activity by regulating the growth, apoptosis and other activities of various cancer cells *in vitro* and *in vivo*. Hence, long-term therapy with appropriate peptide analogs may be able to diminish or stop tumor growth *in vivo (* 45). In advanced cancers, peptide-based vaccines have been used to deliver tumor antigen epitope antigens to T cells in order to elicit a cell-mediated anti-tumor response and improve overall patient survival (46).

### 3.1 Targeted therapy based on peptide ligands

**Figure 1:**
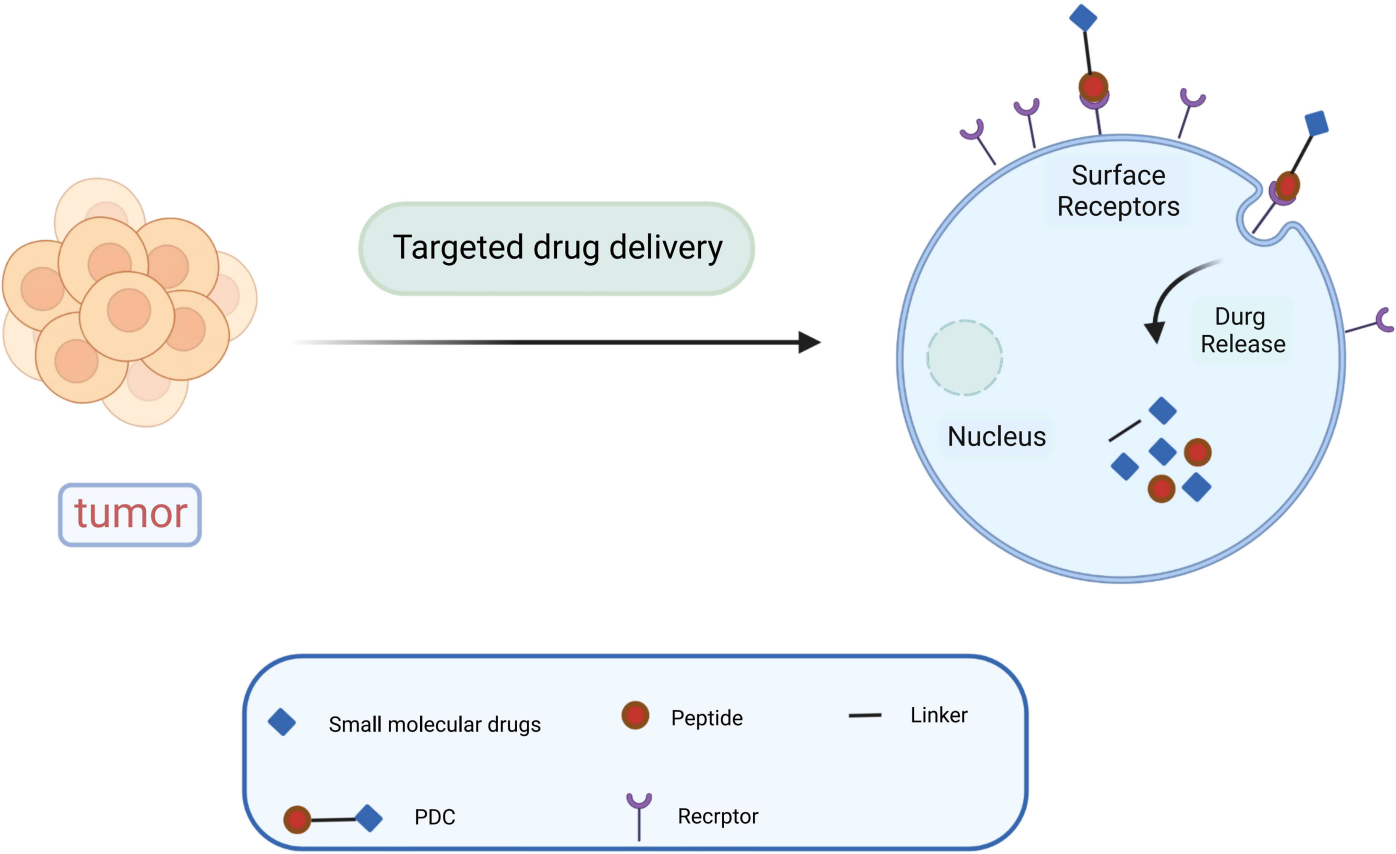
Diagram of peptide-targeted drug delivery.

Paclitaxel combined with platinum-based chemotherapy is the first-line regimen for ovarian cancer. However, the low selectivity and short retention period of current chemotherapeutic agents limit their exposure time at the lesion site (47). Reasonable targeting can significantly improve the treatment effect of ovarian cancer, reduce adverse reactions and drug resistance, and enhance the bioavailability of chemotherapy drugs (48). Anticancer drugs are attached to peptide carriers *via* biodegradable linkers, and peptide are internalized by binding to specific receptors, which in turn deliver the drug to the target site for action (**Figure 1**). In specific tumor-targeted therapies, the use of polypeptides as targeted ligands exhibits a variety of advantages, including low toxicity, low or no immunogenicity, and high specificity (49). Peptides are also highly permeability because they are tiny and generally hydrophilic molecules, allowing for simple and quick access into tumor locations. Polypeptides coupled to the surface of nanoparticles can mediate active targeting, as a complementary strategy to enhance permeability and retention (EPR) effects, and to facilitate targeted cell uptake of drugs (50). A variety of peptide ligands including LHRH peptides, FSHR peptides, RGD peptides, OA02 peptides have been designed and explored for OC targeted therapy (**Table 2**), and drug delivery is achieved through various carrier systems such as liposomes, polymers, micellars, and nanoparticles.

**Table 2:** Peptide ligand functionalized carriers for targeted therapy.

Target	Peptide	Drug/Carrier	Cell	Application Phase	Ref.
LHRHR	LHRH	CPT/PEG	A2780	Experimental Phase	(51)
		DOX/Nanoparticles	OVCAR3	Experimental Phase	(52)
		PTX/Nanoparticles	SKOV3-TR30	Experimental Phase	(53)
		DDP/-	A2780	Experimental Phase	(54)
FSHR	FSH (33-53)	PTX/Nanoparticles	SKOV3	Experimental Phase	(55)
		Gro-αshRNA/Nanoparticles	HEY	Experimental Phase	(56)
	FSH (81-95)	PTX/Nanoparticles	Caov3	Experimental Phase	(57)
αvβ3	cRGDfK	GEM/PLGA nanoparticles	SKOV3	Experimental Phase	(58)
		CBP/unimolecular nanoparticle	OVCAR3	Experimental Phase	(59)
α3	OA02	PTX/micellar nanoparticles	SKOV3/ES2	Experimental Phase	(60)
	cDGWGPNc	Vol/Polymersomes	SKOV3	Experimental Phase	(61)
EGFR	GE11	Dox/Polymersomes	SKOV3	Experimental Phase	(62)
TFR	T7	PTX/Liposomal	A2780	Experimental Phase	(63)
		Seliciclib/PLGA-PEG-NPs	SKOV3	Experimental Phase	(64)

#### 3.1.1 Targeted LHRH peptide receptor therapy

High gonadotropin levels are considered one of the main etiological assumptions for the development of ovarian cancer (65). In a retrospective study, 54.5% FSHR and 88.3% GnRHR expression were detected in 875 cases of high-grade serous ovarian cancer tissues (66). As a result, it has the potential to be a therapeutic target for boosting anti-tumor activities and decreasing adverse effects. LHRH peptides, as a targeted component, allow anticancer medications to be directed specifically to tumors while minimizing injury to normal tissues (51). Recent research indicated that various cytotoxic drugs, such as different combinations of platinum, Doxorubicin (DOX), camptothecin (CPT) with LHRH peptides, had sound drug delivery effects (52). Dharap and colleagues (52) targeted ovarian tumors by combining LHRH peptide with chymopapain (CPT, an apoptosis inducer). They discovered that using LHRH peptide as a targeting moiety improved chemotherapeutic effectiveness, increased tumor apoptosis induction, and reduced the negative effects of CPT on vital organs. Notably, the 2.5 mg/kg dose of CPT-PEG-LHRH in mice did not disrupt the circulating brain and pituitary barriers circulation. Many nanomaterials have been developed as drug carriers to enhance drug penetration and retention effects (67). However, their cellular targeting levels are limited, which limits the toxic effects of drugs (68). LHRH peptide-mediated multifunctional nanomaterials targeting tumors could significantly improve drug delivery and therapeutic efficacy against drug-resistant cancers (69). Pan and colleagues (53) found in a paclitaxel-resistant ST30 cell model that LHRH peptide targeting prolonged drug circulation and enhanced cytotoxicity compared to untargeted nano-delivery vehicles (IC50:3160nM vs 1285nM). LHRH peptide modification of DOX-loaded nanoparticles enhances the specific uptake and therapeutic efficiency of nanoparticles by OVCAR-3 cancer cells (54). *In vitro* studies show 80% cell growth inhibition within 72 hours; *in vivo* imaging results indicated that LHRH-HA-cys-ADOX/Cy5.5 nanoparticles could accumulate at the tumor site and exert long-lasting anti-tumor effects. A recent study discovered that LHRH peptides can directly functionalize novel platinum (IV), increasing platinum water solubility, and that this complex specifically targets the A2780 cell line with 5-8 times more cytotoxicity than LHRH receptor-negative cell lines (70).

#### 3.1.2 Targeted FSHR peptide receptor therapy

Follicle stimulating hormone receptor (FSHR) is another specific hormone receptor for OC. It is expressed not only in plasmacytotic ovarian cancer but also in other particularly aggressive and chemoresistant histological types, such as clear cell and mucinous tumors (71). Peptide sequences on the FSHβ chain have been shown to act as targeting ligands for targeting cells with high expression of FSHR (55). Based on this, Modi’s team (72) designed nanocarriers of FSH (FSH33) peptide-coupled dendrimer molecules as a potential delivery platform. Their study showed that this new system could potentially be used to deliver chemotherapeutic drugs to malignant cells in the ovary while preserving healthy cells. It has been reported that the FSHβ (33-53) peptide has the highest affinity to the FSHR binding domain, and coupling this sequence to PEG-PLA nanoparticles as a paclitaxel (PTX) delivery system to target ovarian cancer cells could enhance the anticancer effects of chemotherapeutic drugs (56). In another work, FSH (33-53) peptide-coupled PEG-PEI copolymer that encapsulated shRNA to silence growth regulatory oncogene alpha (gro-α), successfully down-regulated gro-a expression in HEY cells (FSHR+), which dramatically suppressed cancer cell proliferation, invasion, and migration (57). To achieve higher tumor uptake, Zhang and colleagues (73) used a smaller molecular weight FSH (81-95) peptide as a FSHR-targeting ligand. At equivalent PTX doses, modification (active targeting) of FSH (81-95) peptide improved antitumor efficacy, resulting in a significant delay in tumor growth and 65.57% tumor volume inhibition.

#### 3.1.3 Targeted integrin receptor therapy

The generation of new blood vessels provides oxygen and nutrients for tumor growth and is a hallmark of tumorigenesis and metastasis. It has been demonstrated that the high density of both developing and established vessels contributes to the poor prognosis of ovarian cancer patients (74). Integrins are associated with the process of neovascularization and help to strengthen the adhesion, invasion, and migration of cancer cells (75). Expression of integrin αv subunits was detected in 100% (30/30) solid tumors of ovarian cancer and malignant effusions in 93% (113/121) (76). As a ligand of α_v_β_3_, RGD peptide selectively delivers therapeutic drugs to tumor cells through coupling with liposomes, micelles, nanoparticles and other drug carriers, which enhances the targeted uptake of drugs by tumor cells (58). Kulhari’s team (59) used cRGDfK to modify gemcitabine-containing nanoparticles, which reduced the nonspecific lytic cytotoxicity of the drug compared to unmodified, while enhancing the antiproliferative effect of the drug by modulating mitochondrial membrane potential (DΨm) and reactive oxygen species (ROS) levels. Wang ‘s group (77) similarly demonstrated that in OVCAR-3 ovarian cancer cells, cRGD-coupled nanoparticles exhibited higher cellular uptake (3.4-fold) than non-targeted, thereby leading to higher cytotoxicity. OA02 peptide and cDGWGPNc(A3) peptide are ovarian cancer-specific peptides screened by bacteriophage display technology, and are bound to α3 integrin (60, 78). OA02 peptide modified micellar nanoparticles based on PEG-dendritic bile acid (PEG5k-CA8), enhanced by 6- and 4-fold uptake in SKOV-3 and ES-2 cells, respectively. Moreover, mice treated with 30 mg/kg PTX-OA02-NPs showed the longest survival time, a significant improvement compared to PBS, Taxol, PTX-NPs (61). Polo-like kinase 1 (PLK1) inhibitor volasertib (Vol) is a molecular drug for the treatment of OC patients. A recent experiment conducted by Wang and colleagues (79) demonstrated that the α3 integrin ligand A3 peptide-targeted polymer (A3-Ps) enhanced deep tumor penetration and increased the cycle time of Vol, significantly improving tumor suppression (90%) (90%).

#### 3.1.4 Targeted epidermal growth factor receptor therapy

Epidermal growth factor receptors (EGFRs) belong to the tyrosine kinase family and include four receptor subtypes. EGFR binds to ligands after dimerization, and triggers receptor enzyme activity through phosphorylation of the tyrosine residue at the end of the receptor C-terminal, which causes intracellular signaling to be activated, promotes cell proliferation, inhibits cell apoptosis, and enhances cell motility and angiogenesis (80, 81). EGFR overexpression has been reported in 30-98% of epithelial ovarian cancers (82). Small peptides with EGFR targeting such as LALLT, YHWYGYTPQNVI (GE11) and so on have been screened out in a variety of ways. As EGFR-targeted ligands, GE11 peptides are different from anti-EGFR antibodies and EGF in that they effectively bind to epidermal growth factor receptors without activating the EGFR-mediated signaling pathways (83). Rapid targeting of GE11 peptides can optimize the distribution of some drug carriers (liposomes) in internal tumor tissue to improve *in vivo* efficacy (62). Zou’s team (84) studied the therapeutic effect of GE11 peptide-modified reversibly cross-linked polymersomal doxorubicin (GE11-PS-Dox) on ovarian cancer. They found that GE11-PS-Dox exhibited higher tumor uptake and retention *in vitro* and *in vivo*, effectively inhibited SKOV3 tumor progression and reduced adverse effects in mice compared to control, which in turn significantly increased the survival rate by 100%. This suggests that GE11-guided drug targeting could be an alternative approach to treat EGFR overexpressing ovarian cancer.

#### 3.1.5 Targeted transferrin receptor therapy

Ovarian tumor, particularly high-grade serous ovarian cancer (HGSOC), exhibit enhanced iron uptake and retention. Changes in iron metabolism may promote ovarian cancer cell proliferation and metastasis by manipulating mechanisms such as p53 inactivation and ROS, c-myc expression (85). Transferrin receptor (TFR) is a transmembrane glycoprotein that binds with its natural iron-rich ligand transferrin (TF) to engage in cellular iron transport. TFR1 expression has been reported to be higher in malignant ovarian tissues than in normal ovarian tissues (86). Targeting the TFR of ovarian cancer cells can improve therapeutic drug delivery while also blocking the receptor’s normal activity, resulting in cancer cell death. T7 peptide (HAIYPRH) binds specifically to TFR with high affinity. As a result of the difference in binding sites with TF, the uptake of T7 peptide modified carriers is not inhibited by competition from endogenous transferrin (87). More interestingly, Tf contributes to the targeting of T7 peptides to small cavities on the surface of TfR, which in turn translocate into the cell and increase its endocytosis rate (88).Wu and colleagues (63) reported that T7 peptide can enhance the uptake and penetration of A2780 with higher TFR expression levels to liposomes, thereby significantly enhancing the inhibitory effect of PTX on cells and 3D tumor spheroids. Seliciclib is an inhibitor of cell cycle protein-dependent kinases (CDKs) that plays a potential role in anticancer therapy. Compared to free seliclib, T7 peptide-modified NPs achieved better cellular uptake, effective delivery of seliclib to SKOV3 cells and enhanced cytotoxicity (64). The above results suggest that the improved uptake of drug delivery system by TfR overexpressing cancer cells is achieved by T7 peptide.

### 3.2 Anticancer peptide therapy

Inhibition of tumor growth and metastasis is a strategy for ovarian cancer treatment, which is crucial for the prognosis of patients. Compared with peptides used in drug delivery systems, anticancer peptides are a rising focus in anti-tumor research (89). Anticancer peptides (ACPs) act by inducing apoptosis and disrupting cell membrane structure. Large quantities of evidence signify that cancer cells have higher mobility and more abundant microvilli compared to healthy cells. When ACPs interact with cancer cells, they cause membrane instability, cytotoxicity, and cell lysis (90). In addition, ACPs can also inhibit the proliferation of cancer cells by changing the pH around or inside the cell (91). Small peptides with anticancer effects can be discovered by phage display or chemical synthesis of peptide libraries. We summarize the small peptides that inhibit the growth and metastasis of ovarian cancer cells (**Table 3**), some of which can directly act on ovarian cancer cells to exert anticancer effects. Zhou’s team (92) used a phage display peptide library to isolate the small peptide “SWQIGGN”, which can combine and internalize ovarian cancer cells. It not only inhibited the viability, migration, invasion and adhesion of ovarian cancer cells *in vitro* but also reduced the volume of ascites and controlled tumor growth and metastasis *in vivo*. Another research found the homologous peptide “WSGPGVWGASVK”, identified based on ovarian cancer cell-specific ligand (pc3-1), inhibits adhesion between SKOV-3 cells and HUVECs cells (93) and inhibits cancer cell invasion by down-regulating the active expression of MMP-2 and MMP-9 (94). Furthermore, several short inhibitory peptides, like as “OB3 peptide,” “Smac N7 peptide,” and “A6 peptide,” have been demonstrated to have anticancer effects by acting on ovarian cancer signaling pathways or bioprocess-related targets (95–98).

**Table 3:** Various types of ACPs with their mechanisms.

ACPs	Targets or target cells	Mechanism or function of action	Application Phase	Ref.
SWQIGGN	H08910	Inhibits the expression of VEGF, reduces cell growth and metastasis adhesion	Experimental Phase	(92)
WSGPGVWGASVK	SKOV3	Inhibits adhesion, anti-metastasis	Experimental Phase	(93, 94)
OB3	Leptin	Disruption of leptin-induced proliferation signals by STAT3 phosphorylation and ERα activation	Experimental Phase	(95)
Smac N7	Smac	It down-regulates XIAP and survivin, and up-regulates caspase-3 to promote apoptosis	Experimental Phase	(96)
A6	CD44	Activates CD44 adhesion activity, and inhibits migration and metastasis of CD44-expressing cells	Experimental Phase	(97)
TAT-p + p-8	ABI1	Inhibits invasion and metastasis	Experimental Phase	(98)
PGPIPN	SKOV-3	Reducing the activity of HSF1/HSP70 signaling pathway and inducing cell apoptosis	Experimental Phase	(99)
PNC-27	HDM-2	Binds to MDM-2 and kills cells	Experimental Phase	(100)
LR(LSCQLYQR)	HTS	Stabilizes the double inactive form of the HTS protein, inhibits the growth cells	Experimental Phase	(101, 102)
TAT-D3S2	Beclin1	Binds to Beclin1 and inhibits DIAS3-mediated autophagy, inhibiting cell viability	Experimental Phase	(103)
EP100	LHRHR	Interaction with negatively charged membranes leads to cell lysis and death	Clinical phase	(104, 105)
sv6D	CLEC10A	Induce immune cell proliferation	Experimental Phase	(106)

Unfortunately, due to tumor heterogeneity, drug-resistant phenotypes may emerge late in treatment, including resistance to pro-apoptotic stimuli and/or cytotoxic resistance to anticancer compounds (47). According to certain study findings, bioactive peptides may also have a function in increasing cancer cell susceptibility to chemotherapeutic medicines and reversing cancer cell resistance. Guo’s group (99)found that hexapeptide (PGPIPN), derived from bovine β-casein, significantly increased the sensitivity of DDP to human ovarian cancer cells inhibition of survival and induction of apoptosis *in vitro*. In an *in vivo* model of ovarian cancer, Alagkiozidis’s team (100)discovered that the anticancer peptide PNC-27 could target paclitaxel-resistant cells, and that the addition of PNC-27 to weekly paclitaxel injection greatly decreased tumor development. Similarly, some peptides can be designed to act on anticancer drug targets, and thus provide better inhibitory effect on drug-resistant phenotypes (107). For example, LR peptide (LSCQLYQR) is an anticancer peptide that acts on human thymidylate synthase (hTS), and active site inhibitors of hTS are commonly employed in chemotherapy (101). When LR was given to ovarian cancer cells, it could inhibit intracellular hTS expression. Also, inhibited the growth of approximately 50% of cisplatin-sensitive and resistant ovarian cancer cell lines at low micromolar concentrations (510 μM), more effectively than the same concentration of 5-FU (102).

It is worth noting that although some peptides have inhibitory effects on the growth, invasion and metastasis of ovarian cancer cells. However, due to its weak penetration capacity, it needs to be transported into cells in combination with cell penetrating peptides (CCP) or protein-specific systems to improve the bioavailability of anticancer drugs, and these delivery systems usually do not affect cell proliferation or cell cycle progression (108, 109). D3S2, an autophagy-inhibiting peptide based on the switch II region of DIRAS3, inhibition of autophagy can induce cancer cell death. D3S2 was linked to TAT (a cell-penetrating peptide) to act on OC cells to enhance penetration, and Tat-D3S2 treatment reduced the induction of autophagy in OC cell lines, as evidenced by a decrease in LC3I/LC3II conversion rate (103).EP-100 is composed of a natural ligand for GnRH linked to cleavage peptide (CLIP-71), which delivers the cleaved peptide to GnRH receptor-positive cancer cells, thereby exerting selective toxic effects (104). Kim and colleagues (105) found that EP-100 rapidly destroyed the cell membrane and increased PD-L1 levels after specific binding to ovarian cancer cells, and observed that EP-100 increased anti-tumor effector T cells and IL33 expression and induced anticancer activity *in vivo* when EP-100 and anti-PD-L1 antibodies were combined.

Previous reports have indicated that peptides are generally not toxic in normal host cells and thus may represent a powerful therapeutic tool. Moreover, the combination of anticancer peptides and more aggressive chemotherapeutic agents can improve efficacy, counteract chemotherapy-induced resistance, and reduce overall drug combination toxicity (110). For instance, sv6D acts as a multivalent anticancer peptide that specifically targets the C-type lectin A family 10 member (CLEC10A). In a mouse ovarian cancer model, the peptide sv6D significantly inhibited the formation of ascites when combined with the chemotherapeutic agent paclitaxel or an immunotherapeutic antibody against the receptor PD-1 (106).Combining EP-100 with PARP inhibitor (olaparib) significantly increased the number of nuclear foci of phosphorylated histone H2AX, leading to increased DNA damage in ovarian cancer cells (111). A phase II trial of paclitaxel in combination with EP-100 showed that a subset of patients with target liver lesions showed a greater overall response rate to the combination (69%) compared to paclitaxel alone (16%) (112).

### 3.3 Peptide vaccine treatment

**Figure 2:**
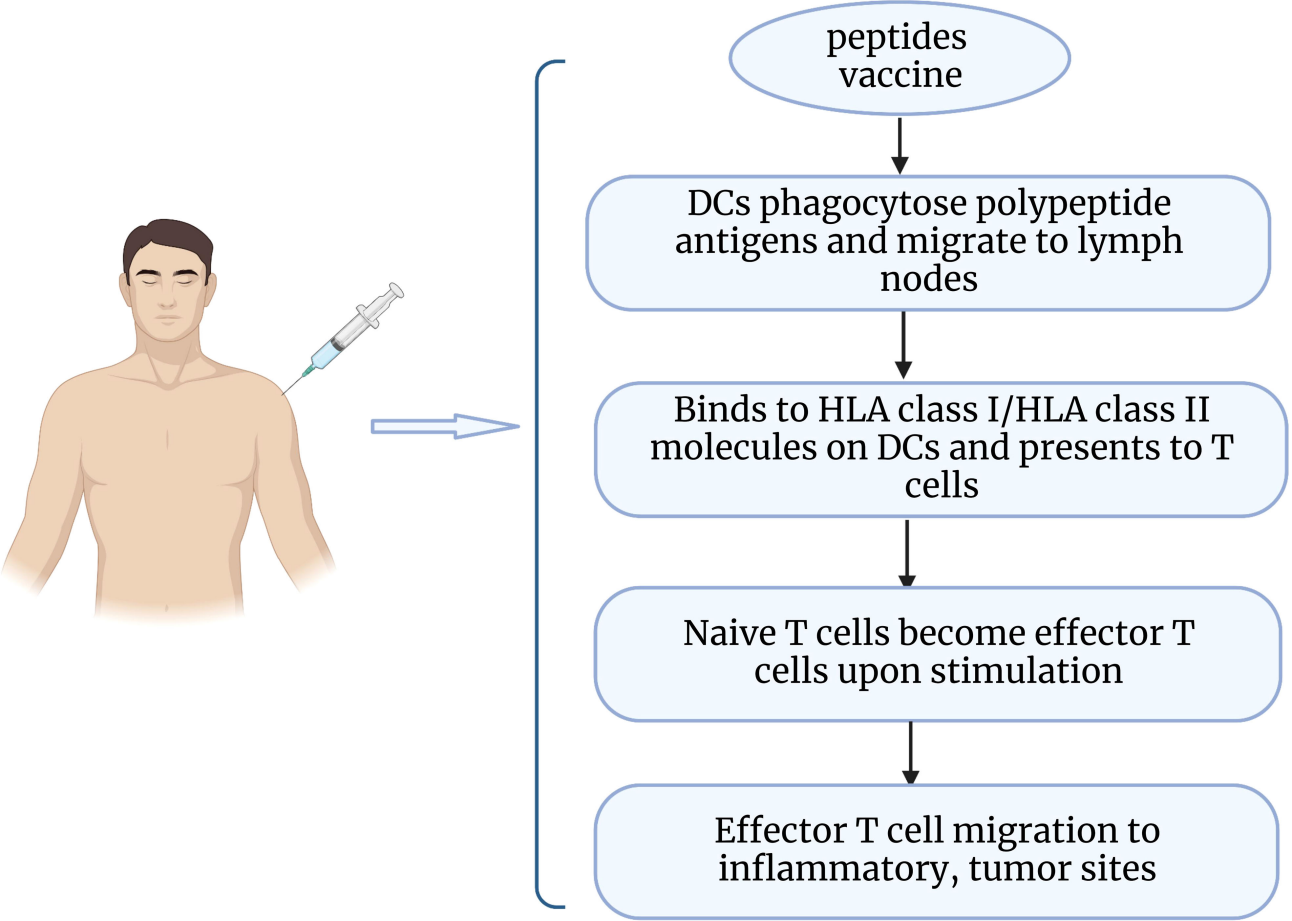
Diagram of peptide vaccine action.

Epithelial ovarian cancer is considered to be an immunogenic tumor. The presence of tumor infiltrating lymphocytes (TIL) in the tumor microenvironment has been shown to increase the 5-year survival rate and improve clinical outcomes for patients (113). As a potential immunotherapeutic route, peptide vaccines are captured by antigen-presenting cells upon entry into the body and delivered to T cells, which then recognize and initiate an immune response that promotes the production of cytotoxic T cells and enhances the body’s ability to fight tumors (114) (**Figure 2**). Peptide vaccines generally consist of an immune adjuvant and a targeted peptide derived from a tumor-associated antigen (TAA) epitope. A retrospective analysis of vaccine treatments over 20 years in ovarian cancer found that peptide vaccines had the highest coverage rate (34.3%), which can be attributed to the fact that peptide vaccines are well tolerated and have fewer side effects (115). A number of peptide vaccines against ovarian cancer are currently in clinical trials (**Table 4**), such as NY-ESO-1 peptide vaccine (116), folic acid receptor α peptide vaccine (120), HER-2/neu peptide vaccine (117), P53 synthetic long peptide vaccine (p53-SLP) (118), and WT1 peptide vaccine (119). Among them, the study of folate receptor alpha peptide vaccines in gynecological tumors has attracted much interest to enhance immunity to folate receptors by producing T cells. E39(EIWTHSYKV) is an HLA-A2 restricted FR peptide that induced a significant, dose-dependent *in vivo* immune response and decreased the risk of recurrence in a clinical phase I/IIa research (124). A recent study identified the optimal vaccination dose of E39 peptide, and the 1000 μg dose significantly improved the 2-year disease-free survival of patients (77.9% at 1000μg vs 31.2% at < 1000μg) (120). However, some researchers believe that overstimulation of E39 may lead to activation induced cell death (AICD). Berry’s group (125) developed the attenuated peptide E39’ (EIWTFSTKV) by replacing two amino acids of E39 peptide and found to elicit an effective CTL response and possibly even avoid AICD caused by repeated stimulation with E39 peptide. The safety of E39’ was subsequently demonstrated in a phase Ib trial (121). This trial also showed that sequential inoculation of 3 times E39 and 3 times E39’ resulted in greater local response and DTH response.

**Table 4:** Various types of peptide vaccines and their immunization mechanisms.

Peptide vaccine	Induction steps	Immunization effect	Application Phase	Ref.
NY-ESO-1	At least five vaccinations at three-week intervals	Vaccine-induced CD8+ and CD4+ T cells remain detectable for 12 months after immunization	Phase I/II	(116)
HER-2/neu	100 ng dose every other day (5 injections in total)	Increased frequency of specific CTL	Experimental Phase	(117)
p53-SLP^®^	Subcutaneous p53SLPVR vaccination 4 times	Induction of p53-specific T cell responses	Phase II	(118)
WT1	Weekly subcutaneous WT1 vaccine for 3 months, then every 2 weeks until disease progression	Increased CD8+ T lymphocytes and specific antibodies	Phase I/II	(119)
E39	Administration of E39 + 250mcg GM-CSF six months of intradermal inoculation	Increased DTH expression	phase I/IIa	(120)
E39’	Arm inoculation with 3 doses of E39 and 3 doses of E39’	Can elicit an effective CTL response	Phase Ib	(121)
FR peptide	Patients were initially treated with low-dose cyclophosphamide, followed by 6 monthly vaccinations for 1 year.	T-cell responses develop slowly, but can last up to 12 months	Phase I	(122)
TPIV200	In 28-day cycles, receiving Durvalumab intravenously on days 1 and 15 of cycles 1-12 and three TPIV200 intradermal injections on day 1 of cycles 1-6.	Produces FRα-specific T-cell responses	Phase II	(123)

Most peptide vaccines are designed based on long-standing tumor-associated antigens (TAA) and have poor immunogenicity. However, peptide vaccines can generate strong immunogenic responses by increasing antigenic epitopes. Multi-epitope vaccines can carry multiple epitopes at the same time and have the ability to be recognized and bound by MHC molecules of multiple genetic backgrounds, resulting in efficient presentation (126). In a phase I clinical trial study, Kalli and colleagues (122) used five putative HLA-A2 class binding peptides (FR30, FR56, FR76, FR113 and FR238) mixed with GM-CSF to form a multi-epitope FR peptide. This experiment showed that more than 90% of ovarian cancer patients produced T-cell immune enhancement and exhibited a stable high immune memory during the observation period. It has been reported that vaccination alone in the early stage of the tumor or under minimal change disease prevents relapse. In contrast, in patients with advanced disease, combination therapy may be more effective in preventing immune escape and gaining clinical benefit (127). Combining peptide-based cancer vaccines with checkpoint medications is a prominent example. Immune checkpoint blockade can restore the body’s immune response by preventing T cell depletion and promoting activation. Zamarin and colleagues (123) evaluated the efficacy of a multi-epitope FR vaccine (TPIV200) in combination with a PD-L1 inhibitor (durvalumab) in patients with advanced platinum-resistant OC. The trial was conducted in 27 patients in 28-day cycles, receiving Durvalumab intravenously on days 1 and 15 of cycles 1-12 and three TPIV200 intradermal injections on day 1 of cycles 1-6. All patients produced a robust FRα-specific T-cell response after treatment. Despite a low overall remission rate, the median overall survival was 21 months, suggesting that combination therapy could help prolong disease control and alter patient outcomes. Likewise, other potential combination therapies such as peptide vaccines and poly ADP ribose polymerase (PARP) inhibitors, anti-angiogenic drugs should be considered.

## 4 Conclusion and future challenges

Despite significant progress in developing new therapies, OC still suffers from adverse treatment reactions and frequent relapses, and there is a great need for new diagnostic and therapeutic approaches. Peptides have shown an important role in the ovarian cancer diagnosis. The differential expression of peptides in body fluids provides a new approach and useful tool used in the early diagnosis of ovarian cancer. The overexpression of some peptide receptors in cancer cells is a potential target for converting peptides into diagnostic tools such as radiolabeled imaging. In addition, surgical resection remains one of the primary treatment options for OC, and fluorescently labeled peptide probes can visualize cancer intraoperatively and provide real-time assessment of margin status imaging. This is critical for increasing surgical success and improving prognosis.

Currently, the field of peptides for tumor targeting and developing specific delivery methods into cancer cells is evolving and playing a strong role in advancing tumor therapy (128). For targeted therapy, peptides are very promising for the delivery of cytotoxic drugs, siRNAs, hormones, fluorophores, or nanoparticles into cells, opening new horizons for targeted therapy as well as combination therapy in metastatic, recurrent ovarian cancer. Although peptides have many advantages in targeted delivery, they also exhibit poor stability and short circulation times in solid tumors. Furthermore, targeted delivery is based on peptide receptor overexpression in tumors, whereas peptide receptor expression in cell lines does not always correspond to the peptide receptor profile in the corresponding human primary tumors and varies greatly between tissue subtypes of the same tumor. An in-depth understanding of ovarian cancer receptor pathogenesis would be beneficial. If we can figure out what controls and regulates the expression of peptide receptors, this could be a new opportunity for the treatment of ovarian cancer.

The discovery of insulin has driven the progress of peptide therapy. Anticancer peptides are characterized by ease of modification, low immunogenicity, and biological and chemical diversity. Peptide-based therapeutics are being tested in clinical trials and it approved by the FDA (129). However, natural peptides are unstable and easily degraded by peptidases *in vitro* and *in vivo*, therefore, some peptides with anticancer effects have limited uptake by tumor cells and usually require administration of large doses (equal to or higher than tolerated) before achieving a therapeutic response. This may cause adverse reactions such as allergy and hemolytic toxicity. Another major challenge faced by anticancer peptides in clinical translation is their poor absorption and low oral bioavailability due to their short half-life and sensitivity to different pH values in the gastrointestinal tract. The conjugation of anticancer peptides with non-structural proteins is thought to improve *in vivo* half-life, but the amino acid number of the peptides must also be considered when selecting conjugates, or effectiveness may be lost due to steric hindrance.

In ovarian cancer, various peptide vaccines have been studied in clinical trials. For chemotherapy-resistant patients, peptide vaccines can effectively improve chemotherapy resistance without serious side effects. In addition, researchers can design personalized peptide vaccines for individual antigenic epitopes. However, peptide vaccines still face some difficulties in clinical application, such as immunosuppressive tumor environment and low immunogenicity. Therefore, more research is needed to improve vaccine-host interactions and to understand the different immune responses to vaccine therapy.

In general, peptide targeted therapies, anticancer peptides and peptide vaccines are difficult to achieve efficacy alone, while combinations with conventional chemotherapeutic agents (carboplatin, docetaxel and doxorubicin), molecular chemotherapeutic agents (PARP inhibitors), and biotherapeutic agents (siRNA and proteins) may yield greater clinical benefit. Recently, the combination of two peptide vaccines targeting different antigenic epitopes on the surface of ovarian cancer has shown promising results. In addition, combinations of peptide vaccines and a number of anticancer peptides are being tested in phase I/II clinical trials. Considering that peptides have shown promising results in the diagnosis and treatment of ovarian cancer patients, combination therapies containing specific targeted peptides, anticancer peptides and peptide vaccines may be more tumor specific, stable, safe and efficacious in the near future. Meanwhile, with rapid advances in computational molecular modeling, machine learning and high-throughput screening technologies facilitating the discovery of new peptides for cancer diagnosis and treatment. In conclusion, peptides can be ideal tools for the diagnosis and treatment of ovarian cancer and are very promising for progressive translation from preclinical development to clinical application.

## Author contributions

LG conceived the topic of the Review. LG, JW, NL, JC and YS contributed to discussions of the content. LG wrote the manuscript. LG, JW, NL, JC and YS corrected and reviewed the article before submission. All authors contributed to the article and approved the submitted version.
